# Early meteorological records from Latin-America and the Caribbean during the 18th and 19th centuries

**DOI:** 10.1038/sdata.2017.169

**Published:** 2017-11-14

**Authors:** Fernando Domínguez-Castro, José Manuel Vaquero, María Cruz Gallego, Ana María Marín Farrona, Juan Carlos Antuña-Marrero, Erika Elizabeth Cevallos, Ricardo García Herrera, Cristina de la Guía, Raúl David Mejía, José Manuel Naranjo, María del Rosario Prieto, Luis Enrique Ramos Guadalupe, Lizardo Seiner, Ricardo Machado Trigo, Marcos Villacís

**Affiliations:** 1 Instituto Pirenaico de Ecología, Consejo Superior de Investigaciones Científicas (IPE-CSIC), Aragón, Zaragoza 50059, Spain; 2 Departamento de Ingeniería Civil y Ambiental, Escuela Politécnica Nacional, Pichincha, Quito 170525, Ecuador; 3 Departamento de Física, Universidad de Extremadura, Badajoz 060071, Spain; 4 Instituto Universitario de Investigación del Agua, Cambio Climático y Sostenibilidad (IACYS), Universidad de Extremadura, 06006 Badajoz, Spain; 5 Grupo de Óptica Atmosférica de Camagüey, Centro Meteorológico Provincial de Camagüey (INSMET), Camagüey 70600, Cuba; 6 Facultad de Ingeniería Eléctrica y Electrónica, Escuela Politécnica Nacional, Pichincha, Quito 170525, Ecuador; 7 Departamento de Física de la Tierra II, Universidad Complutense de Madrid, Madrid 28040, Spain; 8 Departamento de Geología Sedimentarea y Cambio Medioambiental, Instituto de Geociencias IGEO (UCM-CSIC), Madrid, Madrid 28040, Spain; 9 Departamento de Ciencias de la Energía y Mecánica, Universidad de las Fuerzas Armadas (ESPE), Pichincha, Sangolqui 170501, Ecuador; 10 Instituto Argentino de Nivología, Glaciología y Ciencias Ambientales (IANIGLA-CONICET), Mendoza, Mendoza 5500, Argentina; 11 Meteorological Society of Cuba, Commission of History, La Habana, Habana 10400, Cuba; 12 Instituto Riva Agüero, Pontificia Universidad Católica del Perú, Lima 15088, Peru; 13 Instituto Dom Luiz (IDL), Faculdade de Ciências, Universidade de Lisboa, Lisboa 1749-016, Portugal

**Keywords:** Palaeoclimate, Atmospheric science

## Abstract

This paper provides early instrumental data recovered for 20 countries of Latin-America and the Caribbean (Argentina, Bahamas, Belize, Brazil, British Guiana, Chile, Colombia, Costa Rica, Cuba, Ecuador, France (Martinique and Guadalupe), Guatemala, Jamaica, Mexico, Nicaragua, Panama, Peru, Puerto Rico, El Salvador and Suriname) during the 18th and 19th centuries. The main meteorological variables retrieved were air temperature, atmospheric pressure, and precipitation, but other variables, such as humidity, wind direction, and state of the sky were retrieved when possible. In total, more than 300,000 early instrumental data were rescued (96% with daily resolution). Especial effort was made to document all the available metadata in order to allow further post-processing. The compilation is far from being exhaustive, but the dataset will contribute to a better understanding of climate variability in the region, and to enlarging the period of overlap between instrumental data and natural/documentary proxies.

## Background & Summary

The instrumental observation of the atmosphere started at the end of the first half of the 17th century with the invention of the first instruments, such as the thermometer by Galileo. New atmospheric instruments were developed throughout that century and, equally importantly, common measurement scales were implemented allowing the first observational networks to be established, such as the *Accademia del Cimento* between 1654 and 1667^1^. Several short-lived networks in Europe functioned during the 18th century under the auspices of individuals (e.g., James Jurin from 1724 to 1735) or institutions (e.g., the Mannheim network between 1780 and 1795)^2^. However, there were systematic and sustained observations only after the first (1853) and second (1873) International Meteorological Conferences^3^.

Some interesting meteorological observations were performed from the late 17th to the early 19th centuries in Caribbean and central South American countries, including the earliest pressure observations associated with tropical cyclones^4^, eight years of continuous records in Rio de Janeiro^5,6^, the possibly earliest continuous observations above 4,000 masl^7^, and the 1808/1809 observations by Francisco José de Caldas and José Hipólito Unanue in Bogotá and Lima, respectively, which were used to date exactly the unknown eruption of 1809^8^. These early observations were, however, very sparse, discontinuous in time, and often made without homogeneous observation procedures. Moreover, there coexisted different instrumentation and scales. In Spanish America, the prolonged political struggle of the colonies to achieve independence and the reorganization of their administrative services did not favour the continuity of observations^9^. The status of the observations corresponding to the earlier period varies from country to country. Data are usually stored in paper format, preserved in different archives and under different conditions, and most have not been catalogued or digitized.

But these observations can be useful to validate the calibration of natural proxies and meteorological field reconstructions^2,10–12^ or feed historical re-analyses^13^. In recent years there has been a significant effort in the region to increase the number of proxies^14^, most of them being concentrated along the Andean region which is currently poorly covered by instrumental data. These data also help with the analysis of extreme events. Under the LOTRED-SA (Long-Term Climate Reconstruction and Dynamics of South America) initiative, two multi-proxy reconstructions have been produced for temperature and precipitation^15,16^. Given the low data density, further improvement is expected as more proxies become available. The retrieval of early instrumental data is thus crucial in this context because, in conjunction with documentary proxies, they can provide unique information covering wide areas such as the vast South American plains, for which natural proxies are particularly scarce^17^.

An immense quantity of meteorological data in archives and libraries all over the world remains at risk of being lost. The National Meteorological and Hydrological Services (NMHS) have the responsibility of preserving this information, and many countries have promoted data rescue programs. At an international level, such initiatives as the Atmospheric Circulation Reconstructions over the Earth (ACRE)^18,19^ (http://www.met-acre.org/) and the International Data Rescue (I-DARE) Portal (https://www.idare-portal.org/) are trying to unify these data rescue programs and provide them with technical support. It is important to note that most Latin-American NMHS are interested in rescuing meteorological data in their own archives that are often limited to the 20th century, so that the early instrumental data is often still at risk. The LACA&D (Latin America Climate Assessment and Dataset) initiative, in which nine countries collaborate, has been sharing meteorological information since 1900^20^. The ACRE initiative is rescuing earlier data with the aim of lengthening 20th century reanalyses back to the early 19th century. In this regard, there are three ACRE initiatives focused on Latin America—ACRE Chile, ACRE Meso-America (5 countries), and ACRE Argentina. EMERLAC (Early Meteorological Records from Latin-America and Caribbean) will contribute to these ACRE activities.

Finally, it is important to note that all the international data rescue initiatives feed into several international meteorological data repositories, including the International Surface Temperature Initiative (ISTI), International Surface Pressure Databank (ISPD), Global Precipitation Climatology Centre (GPCC), and International Comprehensive Ocean Atmosphere Data Set (ICOADS), among others.

The objective of this paper is to make the EMERLAC dataset available. We have retrieved more than 300,000 meteorological data summarized in 137 series from 20 countries. While we acknowledge that this is not the final word regarding the full extent of data recovery in this area, it is nevertheless a significant step towards improving the availability of the region's early instrumental records.

## Methods

Three steps were followed to create the EMERLAC dataset (Data Citation 1): (i) identifying documentary sources with non-retrieved meteorological data, (ii) digitizing the meteorological data, and (iii) correcting non-systematic biases.

### Finding early meteorological observations

A great diversity of documentary sources are preserved in the region's archives and libraries^17^. The libraries and archives with most potential to preserve early instrumental measurements are those of administrative, academic, scientific, and military institutions.

The present collection was made through a combination of 'in situ' visits to institutions located in the authors' own countries and consultations of Web-based resources. The institutions visited were: *Biblioteca Nacional de España and Archivo Histórico del Real Observatorio de la Armada* (Spain); *Biblioteca Nacional de Portugal and Biblioteca da Academia das Ciencias* (Portugal); *Biblioteca Aurelio Espinosa Pólit* and *Biblioteca del Ministerio de Cultura y Patrimonio del Ecuador* (Ecuador); *Biblioteca de la Sociedad Económica de Amigos del País*, *Biblioteca del Instituto de Literatura y Lingüística*, *Biblioteca Nacional ‘José Martí’*, National Archive and Historical Archive of the *Instituto de Meteorología* (Cuba); *Biblioteca del Servicio Meteorológico Nacional* (Argentina); and *Biblioteca Nacional del Perú* and *Instituto Riva Agüero* (Peru). Fortunately some institutions provide part of their scanned/imaged holdings online. For instance, the *Instituto Histórico e Geográfico Brasileiro* (http://www.ihgb.org.br/acervo1.php), *Biblioteca Nacional Digital* of Brazil (http://bndigital.bn.br/), and *Anales de la Universidad de Chile* (http://www.anales.uchile.cl/) were especially useful in this research. Initiatives such as Google Books (https://books.google.com/) and the Hathi Trust digital library (https://www.hathitrust.org/) are improving the online availability of documentary sources, and were extensively used in this research.

Next, we describe the types of documentary sources consulted in this research so as to provide a general overview of those sources and to serve as a guide in the selection of documentary sources in future research.

**Figure 1:**
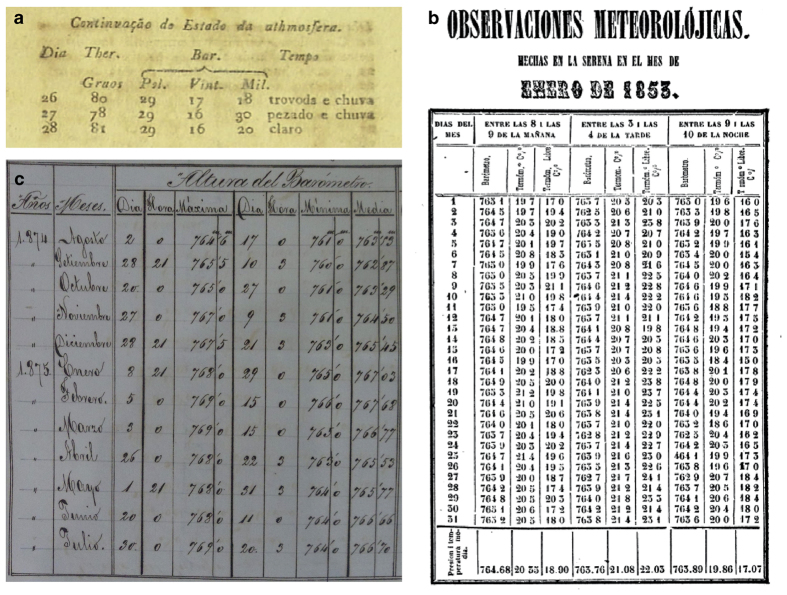
Meteorological measurements recorded in different documentary sources. (**a**) *O Patriota Jornal Literario, Político e Mercantil do Rio de Janeiro*; (**b**) *Anales de la Universidad de Chile*; (**c**) Logbook *Vapor Hernán Cortés* at San Juan (Puerto Rico) (courtesy of the *Archivo Histórico del Real Observatorio de la Armada*, Spain).

1. **Meteorological records published in a monograph by an institution**: These are documentary sources produced by military or scientific institutions that collect together instrumental meteorological measurements. These records usually provide metadata about the instruments used and the methods of observation. Sometimes the records are published with daily (or even sub-daily) resolution, while others only present monthly summaries. These series usually keep the methods of observation fixed even when the observers change. The institutions frequently exchanged their meteorological bulletins, so that these documents are often encountered at specialized libraries far from their original country. There is growing accessibility of these records through online repositories. One example is the *Abstracts from the meteorological observations taken at the stations of the Royal Engineers in the year 1853–1854*^21^ that record observations from the Bahamas and Jamaica.
2. **Newspapers**: Some newspapers during the 18th and 19th centuries contain early instrumental measurements from the previous days or weeks. The usefulness of these records has been examined elsewhere^22^. These series are usually short. The observations appear and disappear from the newspapers without apparent reason. Metadata are infrequent, and the observer and the instruments are usually unknown. It is frequent that some issues of the newspaper are missing or remain only on paper in a specific library or archive. Thus, it is usually difficult to complete long series from newspapers. An example in the present study was the *O Patriota Jornal Literario, Político e Mercantil do Rio de Janeiro* (Fig. 1a).
3. **Scientific annals or proceedings**: These are works presented to an academy, university, or scientific institution. It is quite common for them to provide just summaries of the original meteorological records due to the journal's restriction of space. Such records are usually accompanied by a description of metadata, and some discussion or conclusion inferred from the data. At other times, the original data are presented with regularity but without any metadata or comment. The sources were frequently exchanged with other institutions, which increases the possibility of finding digital copies on Internet. This is the case of the University of Chile with all its annals scanned on the website http://www.anales.uchile.cl/ (Fig. 1b). Many of the data rescued for that country were extracted from there.
4. **Geographical papers**: It was common in the colonies to describe the new territories just discovered. Some of these works provide summaries of the meteorological data recorded or compiled by the author. Metadata are usually scarce. These works usually allow one to identify the earliest observers, and sometimes give clues to finding where the original manuscripts of the early observations are kept. This was the case with the monograph entitled *Viajes científicos a los Andes Ecuatoriales*^23^.
5. **Almanacs**: These are annual publications with information about the agricultural calendar, astronomical ephemerides, and weather forecasts based on traditions or astronomy. Meteorological measurements are infrequent in their records. But *El conocimiento de los Tiempos* presents instrumental measurements from Lima during the period 1754–1856^24^. As with the newspapers, metadata are infrequent, and complete series are hard to obtain.
6. **Navigation records**: Ships' logbooks have also been widely used to understand and reconstruct atmospheric patterns^25–30^. We compiled only records from logbooks of stationary ships. As an example, we found a summary from the logbook of the *Vapor Hernán Cortés* which was anchored in San Juan (Puerto Rico) (Fig. 1c).

To ensure the traceability and reproducibility of the EMERLAC dataset, we have included a detailed reference of the documentary sources consulted in the headline of each series (see the Data Records section). Additionally, a link to an Internet repository has been included when possible.

### Digitization

Due to the high variability in format, layout, typing, and legibility of the documentary sources studied, and taking into account that Optical Character Recognition programs often lead to errors^31,32^, we performed the digitization by key entry. We photographed the documentary sources in situ to have the possibility of re-checking the data when digital copies were not available.

**Figure 2:**
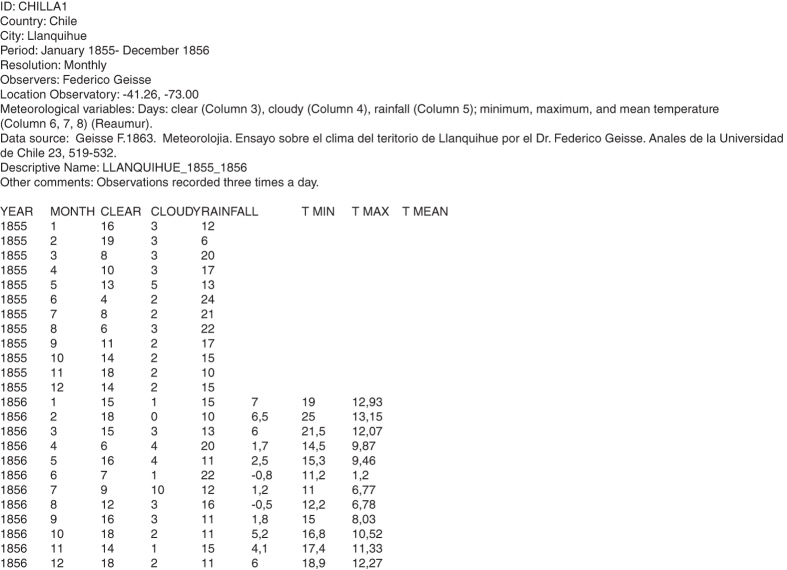
CHILLA1 archive.

All the retrieved data were in numerical or text format. There was no template of a working sheet for the digitization, so each digitizer chose the best option to speed up the process and reduce the reformatting, depending on the format of the original source and taking into account the final structure of the archives (Fig. 2).

The digitizers have climatological knowledge, and are familiar with the variables studied. This allowed for many errors to be corrected during the digitization phase, i.e., number or column transposition, inconsistencies in sequential dates, impossible measurements (e.g., relative humidity above 100%), and the disappearance of (decimal) commas.

The metadata of the series, times of observations, methods of observation, observers, precise location of the observatories, and instruments used were also digitized and provided when available in the original sources. We searched historical literature when metadata were not available in the original sources. The documentary source has been identified in the headline of each series, so that the user can re-check the data if needed.

### Correction of non-systematic biases

The different typology of the retrieved data (time resolution, length, variables) and the lack of close stations at those times made it impossible to perform a systematic and uniform quality control for all the datasets. All the series were subjected to visual inspection to detect transcription mistakes. Of these, there are at least two possible types: one caused in passing from the manuscript to the printed document, and the other during the digitization of these printed sources to the EMERLAC dataset. We detected 488 suspicious values. This is 0.16% of the EMERLA dataset. We corrected 59 values from re-checking the original source, but 429 values were rejected because the suspicious values were coincident with the original source.

A basic quality control was done for the temperature, pressure, and humidity daily series with more than 5 years of observations. These data represent 30% of the total dataset. Each series and variable provides information at different temporal scales. Some series offer 3 observations per day, i.e., morning, afternoon, and night, while others the maximum, minimum, and mean values for each day. The quality control comprised three steps, and was done in the original units:

1. Tolerance test: We flagged all values of each variable above and below the mean plus/minus three standard deviations.
2. Temporal consistency: We computed the difference between consecutive values (the value of the day minus the previous day's). When this difference was greater than 10 °C for temperature, 15 hPa for pressure, and 35% for humidity (or the corresponding figures in the original units), the values were flagged.
3. Internal coherence test: For the series containing maximum, minimum, and mean values, we flagged all the values that did not fulfil the condition maximum>mean>minimum.

No values were corrected or deleted during this process. In total, 90 516 data were evaluated, and only 257 cases (0.28%) were flagged by putting an asterisk after the value. This approach allows any quality control, homogenization, or interpolation processes to be performed by the final users according to their needs.

### Units used in the dataset

The transformation of old units into their contemporary equivalent is not a straightforward process, but usually requires many decisions to be made that may introduce uncertainties. Consequently, we have provided the data in their original units. In this subsection, we provide all the information required to transform old units into current ones.

Temperature measurements: Three scales were used in the dataset, i.e., Celsius, Réaumur, and Fahrenheit. Equation (1) is the formula to transform Réaumur (°R) to Celsius (°C), and equation (2), Réaumur to Fahrenheit (°F).

(1)T(°C)=T(°R)1.25

(2)T(°F)=2.25T(°R)+32

Length units: Different variables were recorded in length units, i.e., pressure (height of the mercury column), precipitation, and evaporation. We found five units of length in the dataset: millimetres, king's feet, French (or Paris) inches, English inches, and Castilian (or Spanish) inches. The conversion factors for French, English, and Castilian inches to mm are 27.0696, 25.3995, and 23.2195, respectively. The measurements were frequently expressed in inches and lines (a line being 1/12th of an inch). The king's feet appear in measurements of Colombia retrieved from the *Semanario de Granada*, with the author giving 1.935 as the conversion factor to mm. This seems to be an error because this is actually the conversion factor from Castilian lines to millimetres, and it is more plausible that the precipitation was recorded in Castilian lines.

Time units: Some meteors, e.g., rainfall, snowfall, hail, fog, are measured in number of days for which that meteor occurs. Others, such as rainfall or sunshine duration, are measured in hours, minutes, and seconds.

Cardinal directions: The wind direction is provided from a compass of 8 or 16 divisions in English or Spanish. Traditional Italianate wind names are not used.

Speed: The wind speed is expressed by adjectives (in the original language) or in km/h.

Most of the series are easily converted with the conversion factors provided. But sometimes the units recorded in the documentary source were wrong, e.g., king's foot. In some cases, too, the units were not recorded in the documentary sources consulted. We then put forward the most plausible unit in the headline of the archive, taking the approach of comparing the documentary data with current measurements in the same or a close-by location. Nevertheless, it is important to bear in mind some caveats about this comparison:

– It is possible that the observations were made in non-standard conditions, such as indoors, influenced by a wall, or partially exposed to solar radiation.
– The metadata rarely explain how the means were computed (daily, monthly, seasonal, or annual).
– It is possible that the barometer measurements were not corrected for temperature, elevation, or standard gravity, especially those of the observations in the first half of the nineteenth century. Moreover, the temperature correction may be done in different ways at this epoch, e.g., the Palatine Meteorological Society adopted 10°R as standard, the Royal Society in London adopted 50°F, and Cotte recommended the freezing point as standard.

## Data Records

In total, we retrieved 301,778 meteorological records from 20 countries of Latin-America and the Caribbean. The earliest observations retrieved are from Lima in 1754, and the latest from La Havana in 1905.

Most of the data are at a daily scale (93.8%), 5.8% of them are monthly, 0.3% annual, and 0.1% seasonal. We recorded all the variables available in every source, the commonest being temperature (104 series), precipitation (67 series), and atmospheric pressure (54 series).

**Figure 3:**
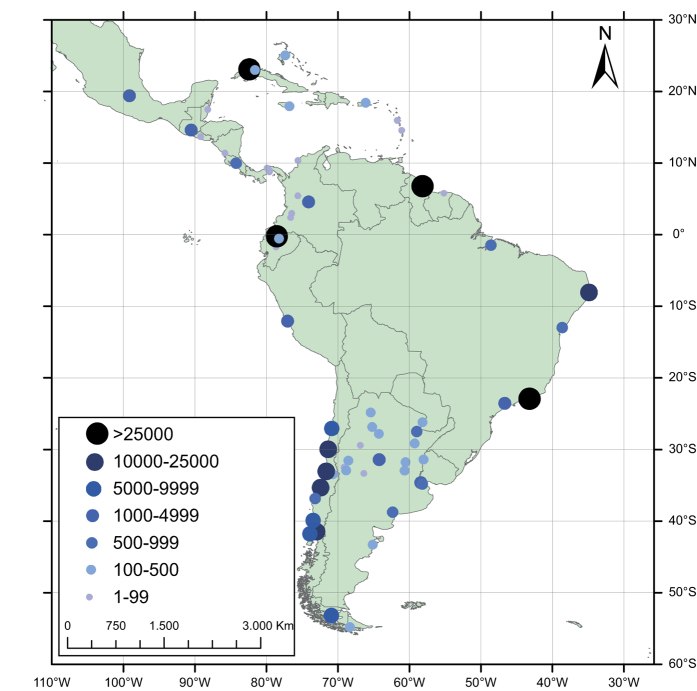
Number of data retrieved by location.

Figure 3 shows the locations of the data retrieved, as well as the availability of data per location. It should be noted that most of the information was retrieved from just four countries—Chile, Cuba, Brazil, and Ecuador (83% of the total).

All the data have been deposited in an appropriate repository (Data Citation 1). The folders are organized by country. In each folder every *.txt* file has been identified with an ID of six letters: the first three refer to the country of the observations, and the last three to the city/location (Table 1). The six letters are followed by a number to differentiate series recorded in the same city/location. Currently the lowest numbers represent the earliest measurements, but this might change if future versions of the dataset are created. Table 2 lists all the series retrieved with their respective archival ID, the period covered, the temporal resolution of the data, and the variables recorded.

To clarify the data and the organization of each series, each archive has a headline with the following information:

ID: Name of the archive as described above.

Country: Current name of the country where the observations were recorded

City: Current name of the city or location where the observations were recorded.

Period: Time period covered by the series, at monthly scale when possible.

Resolution: Time resolution of the series.

Observers: Names of the people who recorded the measurements.

Observatory location: Latitude and longitude of the observatory in WGS84, plus altitude when available. The name of the observatory, or the street where it was located when that location is exactly known. When the precise location is unknown, a probable latitude and longitude is provided.

Meteorological variables: Describes all the meteorological variables recorded, their units, and the corresponding columns in the file.

Data source: The complete reference of the documentary source in which the meteorological record was found.

Descriptive name: A name of the archive that makes reference to the location and the period covered by the series.

Other comments: All the metadata rescued about the observations or the observer. Also, any extreme or rare events recorded by the observer and any other information that could be useful to interpret the series.

After the headline, the first columns give the temporal information of the record (year, season, month, day and hour) and the following columns give the measurements of each meteorological variable. Every column has a short descriptive title. Figure 2 above showed an example of the CHILLA1 archive.

## Technical Validation

As described in the Methods section, we provide raw data. The only correction done had the goal of avoiding non-systematic biases. One must bear in mind that the data are from documentary sources written with very different objectives. The observers had different reasons for recording meteorological observations (scientific, agricultural, navigation, administrative, …), and not only did the care in this recording vary from one observer to another, but, even more so, a given observer might have had greater interest in some meteorological variable than in others.

It also has to be taken into account that, until the end of the 19th century, the observations were not subject to standard rules, so that, methodologically, each series was different.

For all these reasons, we believe that testing the raw data and all the available metadata is the best option with which to optimize the use of the database. Every individual user will be able to apply the type of post-processing that is best suited to their needs.

Some examples of uses that have already been made of part of the dataset are a study to describe the impacts of the 1783–1784 Laki eruption in the Southern Hemisphere^5^, a study of the earliest known continuous 8-year-long instrumental meteorological series for South America^6^, and a study of the earliest known systematic instrumental meteorological observations taken at above 4,000 mamsl^7^.

## Additional Information

**How to cite this article:** Domínguez-Castro, F. *et al.* Early meteorological records from Latin-America and the Caribbean during the 18th and 19th centuries. *Sci. Data* 4:170169 doi: 10.1038/sdata.2017.169 (2017).

**Publisher’s note:** Springer Nature remains neutral with regard to jurisdictional claims in published maps and institutional affiliations.

## Supplementary Material



## Figures and Tables

**Table 1:** Country and city of the series retrieved.

**ID**	**Country**	**City**	**ID**	**Country**	**City**
ARGBAB	Argentina	Bahía Blanca	CHIPAR	Chile	Punta Arenas
ARGBUA	Argentina	Buenos Aires	CHIPHA	Chile	Puerto Hambre
ARGCON	Argentina	Concordia	CHIPMO	Chile	Puerto Montt
ARGCOR	Argentina	Cordoba	CHISCH	Chile	Santiago de Chile
ARGCRT	Argentina	Corrientes	CHIVAL	Chile	Valparaiso
ARGGOY	Argentina	Goya	CHIVLD	Chile	Valdivia
ARGMEN	Argentina	Mendoza	COLALE	Colombia	Alegria
ARGPAR	Argentina	Parana	COLBOG	Colombia	Bogota
ARGRAW	Argentina	Rawson	COLCAR	Colombia	Cartagena
ARGRIO	Argentina	La Rioja	COLMAR	Colombia	Marmato
ARGROS	Argentina	Rosario	COLPOP	Colombia	Popayan
ARGSAJ	Argentina	San Juan	CRISJO	CostaRica	San Jose
ARGSAL	Argentina	Salta	CSRHER	CostaRica	Heredia
ARGSES	Argentina	Santiago del Estero	CUBALQ	Cuba	Alquizar
ARGSJU	Argentina	San Juan	CUBHAV	Cuba	Havana
ARGSLU	Argentina	San Luis	CUBMAT	Cuba	Matanzas
ARGTUC	Argentina	Tucuman	CUBUBA	Cuba	Ubajay
ARGUSH	Argentina	Ushuaia	ECUANT	Ecuador	Antisana
ARGUSP	Argentina	Paramillo de Uspallata	ECULAT	Ecuador	Latacunga
ARGVFO	Argentina	Villa Formosa	ECUQUI	Ecuador	Quito
BAHNAS	Bahamas	Nassau	ECURIO	Ecuador	Riobamba
BELBEL	Belize	Belize	FRABAS	France (Guadalupe)	Basse Terre
BGUGEO	British Guiana	Georgetown	FRAFFR	France(Martinique)	Fort de France
BRABAH	Brazil	Bahia	GUAGUA	Guatemala	Guatemala
BRABEL	Brazil	Belem do Para	JAMUPC	Jamaica	Up Park Camp
BRAREC	Brazil	Recife	MEXMEX	Mexico	Mexico
BRARIO0	Brazil	Rio de Janeiro	NICRIV	Nicaragua	Rivas
BRASPA	Brazil	São Paulo	PANCOL	Panama	Colon
CHIANC	Chile	Ancud	PANGAM	Panama	Gamboa
CHICAL	Chile	Caldera	PANNAO	Panama	Naos
CHICNP	Chile	Concepción	PANPAN	Panama	Panama
CHICON	Chile	Constitucion	PANTAB	Panama	Taboga Island
CHICOQ	Chile	Coquimbo	PERLIM	Peru	Lima
CHICOR	Chile	Corral	PRISJU	PuertoRico	San Juan
CHILLA	Chile	Llanquihue	SALSSA	Salvador	San Salvador
CHIOSO	Chile	Osorno	SURPAR	Suriname	Paramaribo

**Table 2:** Main features of the retrieved series: ID; period covered (periods in italics have important gaps)

**ID**	**Period**	S*	**T**	**P**	**R**	**O**	**ID**	**Period**	S*	**T**	**P**	**R**	**O**	**ID**	**Period**	S*	**T**	**P**	**R**	**O**
ARGBAB1	1860–1882	M	x		x		BRARIO7	1851–1856	M	x				CUBHAV1	1794	M	x	x		x
ARGBAB2	1860–1882	A		x			BRASPA1	1788	D	x	x	x	x	CUBHAV2	*1800–1807*	M	x	x		
ARGBUA1	1801	D	x	x		x	CHIANC1	1871–1872	D	x	x		x	CUBHAV3	1811–1815	M			x	
ARGBUA2	1805	M	x	x	x	x	CHICAL1	1871–1872	D	x			x	CUBHAV4	1825–1831	M	x		x	x
ARGBUA3	1817–1821	A	x		x	x	CHICNP1	1855	D	x		x	x	CUBHAV5	1838–1839	D	x	x		x
ARGBUA4	1822	D	x	x			CHICON1	1871–1872	D	x	x		x	CUBHAV6	1854	D	x			x
ARGBUA5	1822	M	x	x		x	CHICOQ1	1852–1854	M	x				CUBHAV7	1855	D	x			
ARGBUA6	*1805–1887*	S	x				CHICOQ2	1869	D	x			x	CUBHAV8	1854–1856	M			x	x
ARGBUA7	*1856–1888*	M	x		x	x	CHICOQ3	1871–1872	D	x	x		x	CUBHAV9	*1864–1869*	D	x	x		x
ARGCON1	*1875*–*1888*	M			x	x	CHICOR1	1871–1872	D	x	x		x	CUBHAV10	1870–1880	D	x	x	x	x
ARGCOR1	1873–1888	M	x	x	x	x	CHILLA1	1855–1856	M	x			x	CUBHAV11	1885–1905	D	x	x	x	x
ARGCRT1	1873–1887	M	x	x	x		CHILLA2	1856	A				x	CUBMAT1	1837.1846	D	x			
ARGGOY1	1876–1887	S	x				CHIOSO1	1854	M				x	CUBUBA1	1796–1799	M	x			
ARGGOY2	1876–1887	M			x		CHIPAR1	1851	D	x	x		x	ECUANT1	1845–1846	M	x	x	x	x
ARGMEN1	*1875*–*1886*	M	x		x		CHIPAR2	1853–1854	M	x		x	x	ECULAT1	1873–1874	D	x			
ARGPAR1	1875–1882	M	x		x		CHIPAR3	1855–1856	M	x				ECUQUI1	1825–1828	M	x			
ARGRAW1	1881–1887	M	x				CHIPAR4	1859–1859	D	x	x	x	x	ECUQUI2	1864–1865	M	x	x	x	x
ARGRIO1	1875–1878	M			x		CHIPAR5	1859	D	x				ECUQUI3	1864	D	x	x		x
ARGROS1	1875–1888	M			x		CHIPHA1	1822	M	x	x		x	ECUQUI4	1866	M	x	x	x	x
ARGSAJ1	*1867*–*1887*	M	x				CHIPMO1	1859	M	x			x	ECUQUI5	1870–1873	M	x			
ARGSAJ2	1867–1887	S			x		CHIPMO2	1859	A			x	x	ECUQUI6	1878–1881	M	x		x	x
ARGSAL1	*1873*–*1886*	M	x		x		CHIPMO3	1868–1869	D	x	x		x	ECUQUI7	1878–1882	D	x	x	x	x
ARGSES1	1874–1886	M	x		x		CHIPMO4	1871–1872	D	x	x		x	ECURIO1	1872	D	x			
ARGSJU1	1875–1888	M	x		x		CHISCH1	1824–1850	D				x	FRABAS1	1800	M				x
ARGSLU1	1874–1877	M			x		CHIVAL1	1837–1839	M	x				FRAFFR1	1807	M				x
ARGTUC1	1855–1864	M	x				CHIVAL2	1853–1855	M	x				GUAGUA1	1846–1848	M	x			
ARGTUC2	1874–1885	M	x		x		CHIVAL3	1863–1868	D	x			x	GUAGUA2	1847–1848	M	x			
ARGUSH1	1876–1885	M	x		x		CHIVAL4	1871–1872	D	x	x		x	GUAGUA3	1856–1859	M	x	x	x	x
ARGUSP1	1886–1888	M			x		CHIVLD1	1851–1854	M	x				GUAGUA4	1862	M	x	x	x	x
ARGVFO1	1879–1887	S	x	x			CHIVLD2	1851–1852	M	x		x	x	GUAGUA5	1856–1882	A			x	
ARGVFO2	1879–1888	M			x		COLALE1	1808	M			x		JAMUPC1	1853–1854	M	x	x	x	x
BAHNAS1	1852–1854	M	x	x	x	x	COLBOG1	1807	M			x		MEXMEX1	1769	D	x	x		x
BELBEL1	1848–1888	A			x		COLBOG2	1808	D	x	x	x		MEXMEX2	1858	M	x	x		
BGUGEO1	1846–1856	D	x	x		x	COLBOG3	1823–1824	D	x	x		x	NICRIV1	1880–1894	A			x	
BRABAH1	1846	D	x				COLBOG4	1824	M	x				PANCOL1	*1865*–*1886*	A			x	
BRABAH2	1846	M	x				COLBOG5	1848–1850	M	x	x			PANGAM1	1882–1886	A			x	
BRABEL1	1845–1849	M	x	x	x		COLBOG6	1848–1850	M	x		x	x	PANNAO1	1882–1886	A			x	
BRAREC1	1808–1810	D	x	x			COLBOG7	1831–1835	D				x	PANPAN1	1879–1882	A			x	
BRAREC2	1842–1843	D	x	x	x	x	COLBOG8	1831–1835	D	x	x			PANTAB1	1861–1864	A			x	
BRAREC3	1842–1842	M	x	x	x	x	COLCAR1	1808	M			x		PERLIM1	1754–1856	A	x			
BRAREC4	1842–1843	D	x	x	x	x	COLMAR1	1827	M			x		PERLIM2	*1791*–*1794*	D	x			
BRARIO1	1781–1788	M	x	x	x	x	COLMAR2	1853–1854	M			x		PERLIM3	*1799*–*1800*	D	x			
BRARIO2	1781–1788	D	x	x	x	x	COLPOP1	1808	M			x		PRISJU1	1874–1875	M	x	x		
BRARIO3	1813–1814	D	x	x		x	CRISJO1	1866–1867	M		x	x	x	SALSSA1	1889–1892	A			x	
BRARIO4	1821–1822	D	x	x			CRISJO2	1866–1880	A	x		x	x	SURPAR1	1851–1854	M	x			
BRARIO5	1836–1837	M	x	x		x	CSRHER1	1866–1867	M	x		x	x							
BRARIO6	1844	D	x	x		x	CUBALQ1	*1821*–*1824*	M			x								

*scale (A: annual, S: seasonal, M: monthly, D: daily); variables recorded—temperature (T), atmospheric pressure (P), rainfall (R), and other variables (O).
